# Beware of Mycoplasma Anti-immunoglobulin Strategies

**DOI:** 10.1128/mBio.01974-21

**Published:** 2021-11-16

**Authors:** Yonathan Arfi, Carole Lartigue, Pascal Sirand-Pugnet, Alain Blanchard

**Affiliations:** a Univ. Bordeaux, INRAE, Biologie du Fruit et Pathologie, UMR 1332, Villenave d’Ornon, France; Ohio State University

**Keywords:** mycoplasma, immunoglobulin, evasion, persistence, immune evasion

## Abstract

Mycoplasmas are small, genome-reduced bacteria. They are obligate parasites that can be found in a wide range of host species, including the majority of livestock animals and humans. Colonization of the host can result in a wide spectrum of outcomes. In many cases, these successful parasites are considered commensal, as they are found in the microbiota of asymptomatic carriers. Conversely, mycoplasmas can also be pathogenic, as they are associated with a range of both acute and chronic inflammatory diseases which are problematic in veterinary and human medicine. The chronicity of mycoplasma infections and the ability of these bacteria to infect even recently vaccinated individuals clearly indicate that they are able to successfully evade their host’s humoral immune response. Over the years, multiple strategies of immune evasion have been identified in mycoplasmas, with a number of them aimed at generating important antigenic diversity. More recently, mycoplasma-specific anti-immunoglobulin strategies have also been characterized. Through the expression of the immunoglobulin-binding proteins protein M or mycoplasma immunoglobulin binding (MIB), mycoplasmas have the ability to target the host’s antibodies and to prevent them from interacting with their cognate antigens. In this review, we discuss how these discoveries shed new light on the relationship between mycoplasmas and their host’s immune system. We also propose that these strategies should be taken into consideration for future studies, as they are key to our understanding of mycoplasma diseases' chronic and inflammatory nature and are probably a contributing factor to reduce vaccine efficacy.

## INTRODUCTION

## MYCOPLASMA: SMALL PARASITES CAUSING LONG-LASTING PROBLEMS

The colloquial term “mycoplasmas” refers to a number of bacteria belonging to the *Mollicutes* class and the *Mycoplasma* and *Ureaplasma* genera. They are characterized by their small genomes (0.6 to 1.35 Mb) derived from a common ancestor with *Firmicutes* through drastic gene losses resulting in a lack of cell wall and limited metabolic capacities (for a review, see reference 1). Owing to these deficiencies, mycoplasmas are obligate parasites that rely on their hosts for the production of a large array of essential metabolites (2, 3). They have been identified in a wide range of vertebrate hosts, including multiple livestock and wild animal species and humans. Horizontal gene transfer events occur frequently among mycoplasmas sharing a common host, partially counterbalancing their genome erosion (4).

Some *Mycoplasma* species are found in the bacterial flora of healthy, asymptomatic individuals. This observation suggests that they can be highly successful parasites, managing to be sufficiently discrete to remain undetected by their host and not triggering the immune system.

In stark contrast, many *Mycoplasma* species are pathogenic for their host. Intriguingly, this is also true for species that are known to be carried asymptomatically. Factors explaining this dual nature are currently unknown. Infection by mycoplasmas can result in a wide range of clinical outcomes, depending on the species, the infected individual, and the occurrence of other coinfections (5–7). While some mycoplasma diseases can be acute, the most frequent scenario is a chronic inflammatory disease with high morbidity and low mortality such as arthritis, urethritis, or walking pneumoniae. Interestingly, the majority of *Mycoplasma* species do not secrete toxins, and pathogenicity appears to be due, in a large part, to the host inflammatory reaction (8) and to cellular damages associated with the bacterial production of reactive oxygen species (9, 10). Stimulation of inflammation is not related to typical pathogen-associated molecular patterns (PAMPs) (e.g., peptidoglycan, lipoteichoic acid, flagellin, and classical lipopolysaccharides), as mycoplasmas are devoid of these molecules (11, 12). They do, however, present lipoproteins and lipopeptides in their membranes that have been shown to promote inflammation through binding to Toll-like receptors (13, 14).

Once established, most mycoplasma infections become chronic, despite a seemingly normal immune response during which both cell-mediated and humoral mechanisms are involved (15–18). The antibody response to mycoplasma infections follows a typical development kinetic: specific IgM is detected within the first week of infection, followed by the production of high titers of specific IgG and IgA (15, 19, 20). While, in many cases, the immune system is able to control the infection and reduce the bacterial load, significant populations of mycoplasma can be maintained in colonized tissues (21–24). This observation suggests that the local immune response is more relevant to immunity and clearance than the systemic response, in particular, through the localized production of high antibodies titers in the mucosa.

Currently, there are limited options for the prevention or treatment of infections by mycoplasmas. Antibiotics treatments are available, but infections often reoccur after treatment, and emerging resistant strains have been described for most antibiotics in the majority of mycoplasmas (25). No vaccine is available for human-pathogenic species, and only a small number of low-efficiency vaccines are available for livestock-pathogenic species (26). Most studies report that vaccination yields a typical immune response with high titers of circulating specific antibodies, followed by an often disappointing level of protection when vaccinated individuals are challenged with pathogenic strains (26–29). This is intriguingly reminiscent of what can be observed during natural infections, with a correct onset of the immune response followed by an eventual failure to clear the pathogen.

## MYCOPLASMAS HAVE EVOLVED MULTIPLE PASSIVE STRATEGIES TO EVADE ANTIBODIES

**FIG 1:**
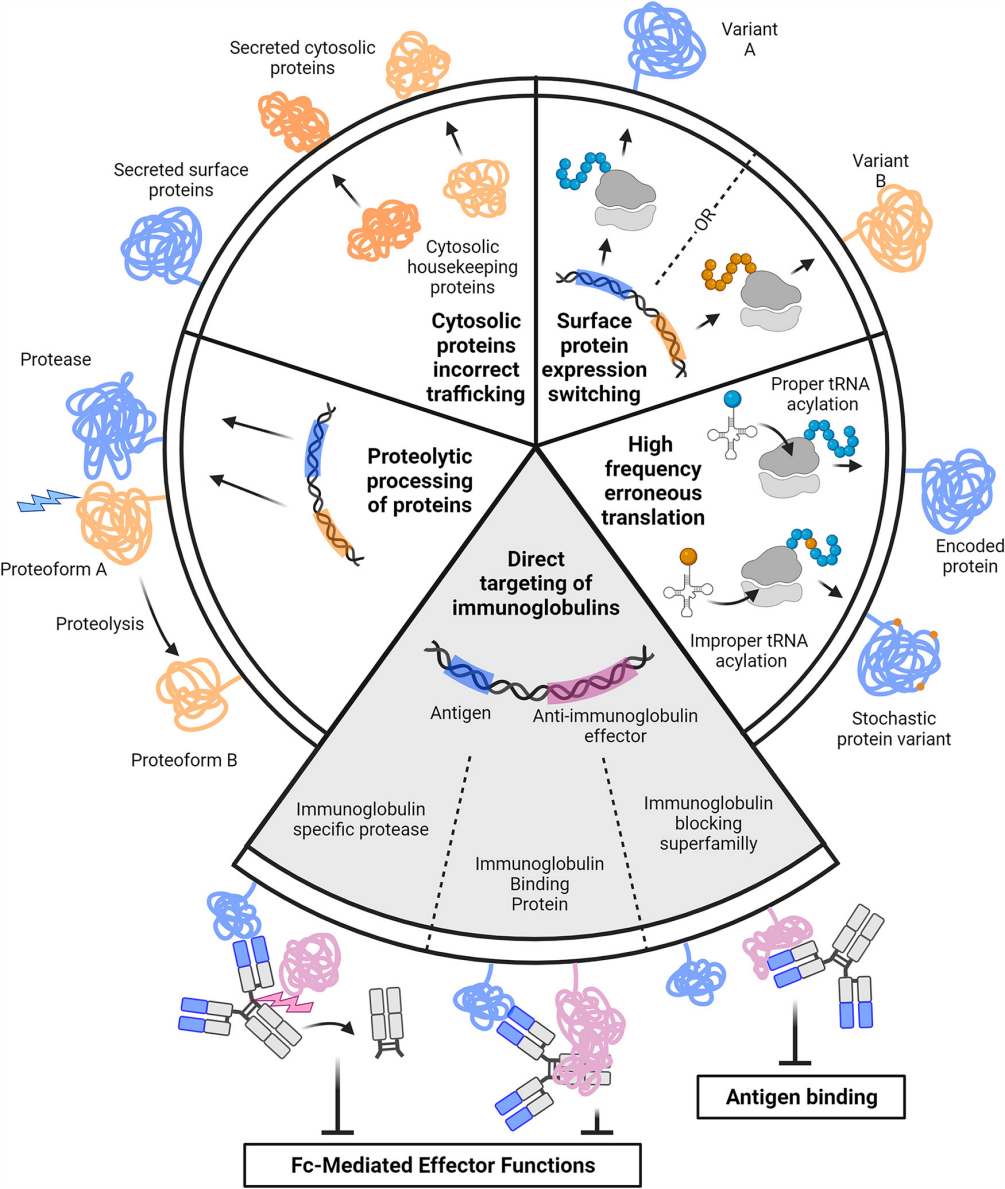
Overview of the antibody evasion strategies described in mycoplasmas. Each segment corresponds to a schematic representation of an antigenic variation mechanism (white segments) or an antibody-targeting mechanism (gray segment). This figure was created with BioRender.com.

Immune evasion, i.e., the ability of an organism to subvert and exploit the immune mechanisms of its host (30), is a key question in mycoplasmology and has been the subject of numerous studies (31, 32). Of particular interest is the ability of mycoplasmas to successfully thwart their host’s humoral immune response, as these bacteria can infect recently vaccinated individuals and persist even in the presence of high titers of specific antibodies (21, 22, 33). Over the years, multiple mechanisms and effectors have been characterized, indicating that mycoplasmas have evolved complex suites of strategies geared toward limiting the efficiency of the antibody response (Fig. 1).

The majority of the known mechanisms give mycoplasmas the ability to significantly alter the composition of their surface over time and thus generate important antigenic diversity (34, 35).

First, many mycoplasmas present multiple genomic loci encoding variants of major and immunodominant lipoproteins (36) and are able to switch expression between these loci. Switching can be the result of different genetic mechanisms (DNA slippage, site-specific recombination, reciprocal recombination, and gene conversion) that lead to either on/off switching of expression, size variation of the expressed protein, antigenic masking, or domain shuffling (for a review, see reference 37). This variation has been described *in vitro* but also *in vivo* during natural infection (38–41). Switching from one variant to the other appears to be stochastic, and antibodies targeting the variable surface proteins provide a negative selection pressure by inhibiting growth of mycoplasmas expressing a specific lipoprotein variant (42, 43).

Second, recent studies suggest that the selective pressure of the parasitic lifestyle has led mycoplasmas to maintain deficient pathways, seemingly in exchange for antigenic variability. For instance, mycoplasmas are the only known bacteria to harbor editing-defective aminoacyl-tRNA synthetases (AARSs). As they are devoid of proofreading activity, these enzymes allow acylation of a wrong amino acid on the tRNA, a process which is normally subject to strict quality control (44–46). In the case of Mycoplasma mobile, it was shown that the incorporation of an improper amino acid occurs at an estimated frequency of 1 in 200, when a typical error rate of 1 in 3,000 is seen for nondefective AARSs. This leads to frequent mistranslation (i.e., incorporation of an incorrect amino acid in the polypeptide chain) and therefore yields a highly polymorphic and stochastic proteome. Another example involves the incorrect trafficking at the cell surface of proteins that are normally cytoplasmic. Studies of the surface proteomes of Mycoplasma hyopneumoniae and Mycoplasma pneumoniae have shown that these improperly surface-exposed proteins could account for almost half of the surfaceome (40% and 58%, respectively) (47, 48). These normally cytoplasmic proteins have been shown to moonlight as adhesins interacting with receptors on the surface of host cells and components of the extracellular matrix (ECM) (47–53). Therefore, in addition to increasing the complexity of the surface proteome, they could also serve the ancillary function of camouflaging the mycoplasma cell by aggregating host components at its surface (54).

Finally, further diversification of the mycoplasma surface antigen repertoire is caused by a mechanism of posttranslational protein cleavage. This processing phenomenon was first described in the swine respiratory pathogen M. hyopneumoniae, for which about half of the detectable proteins found on the cell surface were targets of proteolytic processing events (55). Similar protease activities targeting surface antigens were also found with the human respiratory pathogen M. pneumoniae (56). Many processed surface proteins have putative functional interactions with key host extracellular matrix molecules, indicating that protein function, pathogenesis, and, ultimately, the development of efficacious vaccines are influenced by proteolytic processing events that remain ill-defined (55–57).

## MYCOPLASMAS DIRECTLY TARGET THE HOST ANTIBODIES

In addition to the strategies described above aimed at generating an elusive and ever-changing antigenic target, mycoplasmas have also evolved mechanisms geared toward the direct targeting and inactivation or destruction of the host antibodies (Fig. 1).

Historically, one of the first mechanisms to be described is based on the production of proteases targeting the hinge region connecting the Fab and Fc domains of the antibody. The action of these proteases leads to the removal of the Fc fragment, which, in turn, prevents the activation of Fc-mediated processes such as complement recruitment and opsonization (58–60).

In *Ureaplasma* species, it was shown as early as 1984 that a serine protease was dedicated to cleaving the hinge region of IgA1 (61, 62). Unlike other IgA1 proteases of pathogenic bacteria that colonize mucosal surfaces such as Haemophilus influenzae, Neisseria meningitidis, and N. gonorrhoeae, the IgA1 protease of ureaplasmas is not secreted but located at the cell surface. It does, however, share the same cleavage site, located between the proline and threonine residues 235 and 236 of the immunoglobulin alpha heavy chain (63, 64). Interestingly, despite the fact that *Ureaplasma* genomes have been sequenced 20 years ago, the gene encoding this IgA1 protease activity remains unknown.

More recently, the protease CysP was identified in the avian pathogens Mycoplasma gallisepticum and Mycoplasma synoviae and characterized as a surface-exposed IgG-specific cysteine protease that cleaves antibodies into Fab and Fc fragments (65). The genes encoding CysP are considered to have been shared by horizontal gene transfer between M. gallisepticum and M. synoviae due to their high degree of conservation (∼88% amino acid sequence identity) in two mycoplasmas that are not phylogenetically related (66). Interestingly, homologous sequences, albeit with much lower percentages of identity, have also been identified in other avian mycoplasmas such as Mycoplasma glycophilum, Mycoplasma gallinarum, Mycoplasma gallinaceum, Mycoplasma gallopavonis, Mycoplasma pullorum, and Mycoplasma meleagridis. However, the function of these genes as encoding IgG proteases has yet to be confirmed.

**FIG 2:**
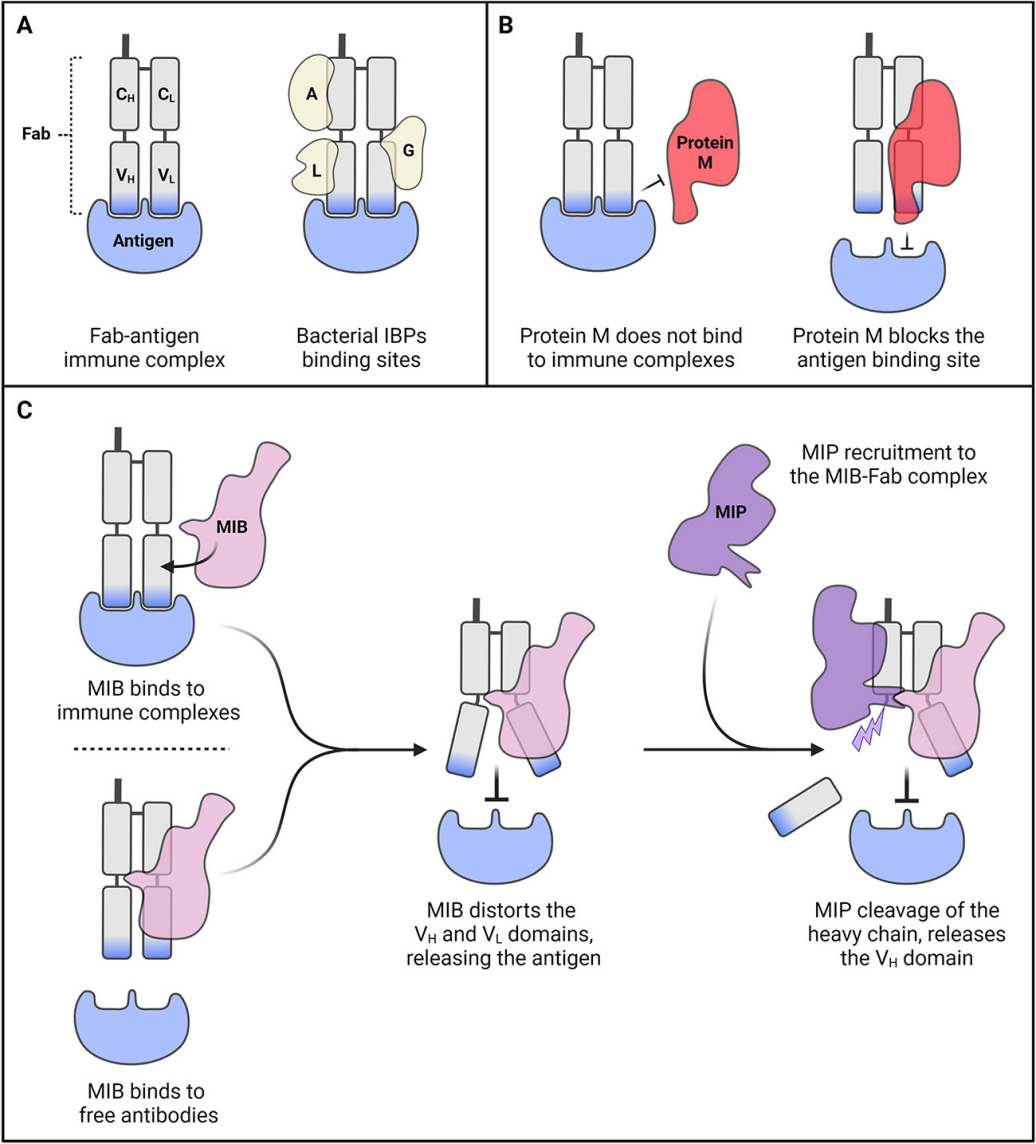
Schematic overview of the immunoglobulin-binding mechanisms found in mycoplasma. (A) Known bacterial immunoglobulin-binding proteins such as protein A, protein L, and protein G (yellow; noted A, L, and G, respectively) can bind to various domains of the Fab fragment of the antibody (gray), irrespective of the presence or not of a bound antigen (blue). (B) Protein M (red) is only able to interact with free antibodies, as its binding is hindered by the presence of the antigen. (C) MIB (pink) is able to bind to both free antibodies and immune complexes. Upon binding, it displaces the V_H_ and V_L_ domains, resulting in either the release of the previously bound antigen or in inhibition of antigen binding for free antibodies. Subsequently, MIB recruits the protease MIP (purple), which definitively inactivates the antibody through proteolytic cleavage. This figure was created with BioRender.com.

In addition to antibody degradation by proteolysis, strategies based on immunoglobulin-binding proteins (IBP) have also been discovered in mycoplasmas. IBPs are found in multiple bacterial pathogens, and their function is to interact tightly with the antibody Fc or Fab domains. Although they do not impact the interaction with the antigen (Fig. 2A), they often prevent the recognition of the antibodies by other proteins and thus can block the antibody-mediated effector functions (67, 68). First evidence for the presence of IBPs in mycoplasmas came in 1991, when Lauerman et al. showed that two proteins in M. synoviae (∼80 and ∼90 kDa) are able to bind to the Fc part of chicken IgG (69). Later on, in 2009, Moussa et al. have shown that a major lipoprotein from Mycoplasma penetrans (∼38 kDa) is able to bind to human serum IgA, but not IgG (70). It should be noted that the gene encoding this antigen belongs a family of genes that are subjected to phase variation linked to a site-specific tyrosine recombinase that mediates inversion of promoter-like sequences (71, 72).

**FIG 3:**
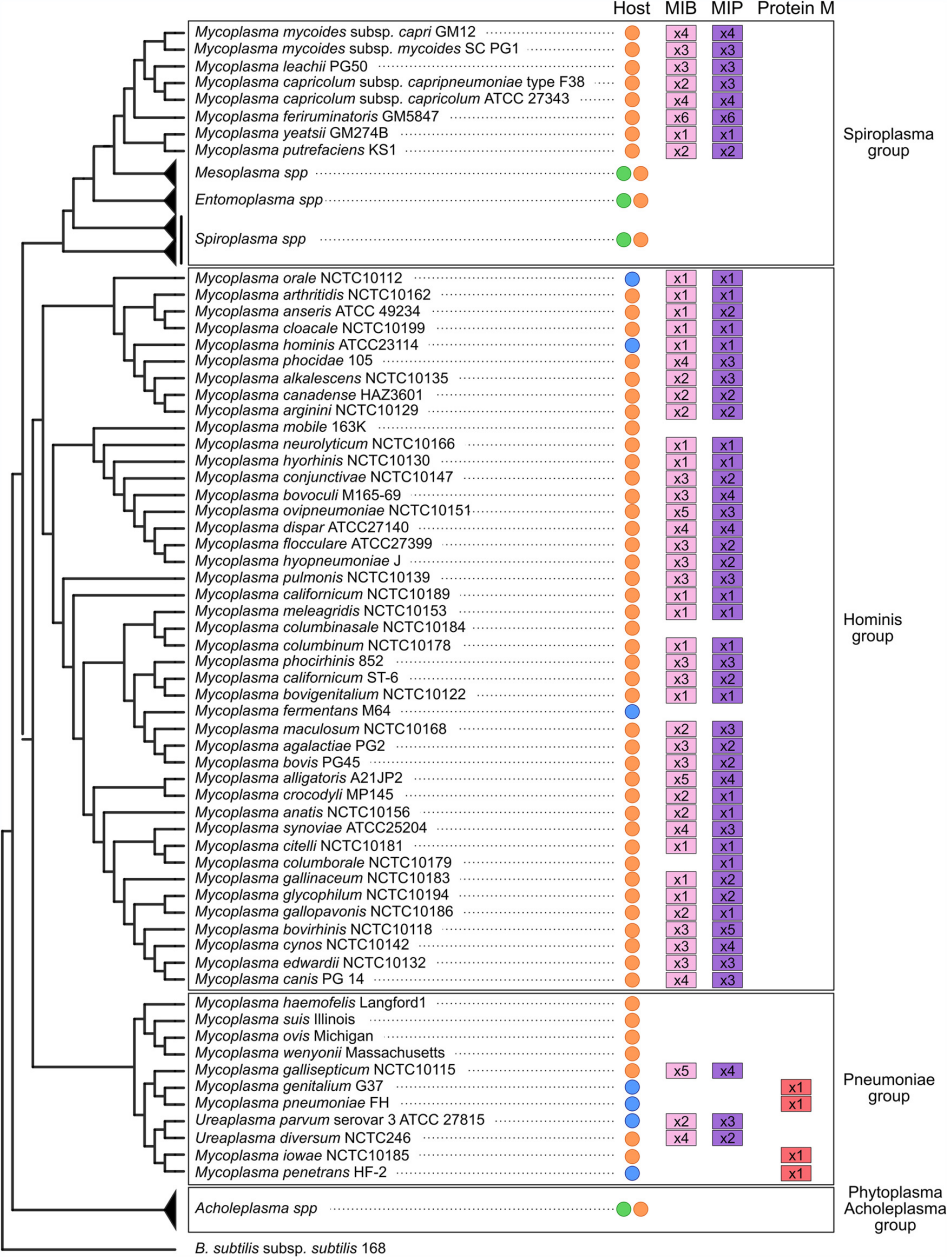
Distribution of immunoglobulin-blocking superfamily homologs in *Mollicutes* genomes. A phylogenic tree of representative *Mollicutes* species was generated. For each species, the host is given as a colored circle (orange, animal; blue, human; green, plant), and the presence in the genome of predicted IBP homologs is given as a colored box (pink, MIB; purple, MIP; red, protein M). The numbers in the colored boxes indicate the number of MIB, MIP, and protein M paralogs found in a given species. The phylogenetic tree and data presented in this figure were extracted with permission from Nottelet et al. (76).

Recently, a novel group of mycoplasma-specific IBPs termed “immunoglobulin blocking superfamily” has been discovered and extensively characterized (Fig. 3). In 2014, Grover et al. reported the identification of “protein M,” a 62-kDa surface protein encoded by the locus MG281 of Mycoplasma genitalium, which is able to bind with a very high affinity to the light chains of various immunoglobulins (IgG, IgA, and IgM) (73). *In vitro* analysis and structural characterization have highlighted that the C-terminal domain of protein M forms a cap that partially sits on the antigen-binding site and, as a result, blocks the antibody-antigen interaction. This ability to prevent the formation of immune complexes was not described before for any other bacterial IBPs. Interestingly, it was demonstrated that protein M is not able to interact with preestablished immune complexes and thus acts preemptively on free antibodies (Fig. 2B). Currently, the impact of protein M on Fc-mediated effector functions is unknown and has not been evaluated in the study describing this system. Searches for protein M homologs in available *Mollicutes* genomes yielded only a small number of hits. A highly similar homolog was identified in the closely related species Mycoplasma pneumoniae. Encoded by MPN400 and termed IbpM, this protein was shown to be functionally identical to protein M by Blötz et al. in 2020 (74). In addition, distant homologs have been predicted in Mycoplasma iowae, M. gallisepticum, M. penetrans, Mycoplasma tullyi, Mycoplasma imitans, and Mycoplasma pirum. Protein M therefore appears to be found only in members of the *Pneumoniae* group (Fig. 3) but is noticeably absent from the intracellular hemotropic species such as Mycoplasma suis or Mycoplasma haemofelis.

Interestingly, in 2016, we have characterized a new anti-immunoglobulin system which couples both an IBP and an antibody-specific protease in the goat pathogen Mycoplasma mycoides subsp. *capri*. It is based on two surface proteins, mycoplasma immunoglobulin binding (MIB) and mycoplasma immunoglobulin protease (MIP) (75). MIB is a distant homolog of protein M and shares its main function, binding tightly to the antibody light chain. Upon interaction with the immunoglobulin, MIB is able to recruit the serine protease MIP and to activate its catalytic activity. MIP cleaves the antibody heavy chain at a site located on the linker connecting the V_H_ and C_H_1 domains. Following proteolytic cleavage, the V_H_ domain is detached from the rest of the antibody, which is no longer able to target its cognate antigen due to the loss of half of its complementarity-determining regions (Fig. 2C). The genes encoding MIB and MIP homologs are found in most animal-pathogenic *Mollicutes* (Fig. 3), and most species carry multiple copies of MIB and MIP. Characterization of these paralogs in M. mycoides subsp. *capri* indicated that they are functionally redundant and cross-compatible, suggesting that their sequence diversity could be another component of antigenic variation (76).

Protein M and MIB share the same basic function, and their IBP activities are mediated through a similar protein structure despite having drastically different amino acid sequences. MIB appears to be an enlarged version of protein M, with a common core fold decorated by a large MIB-specific N-terminal arm domain. This arm domain appears to be key to the interactions with the protease MIP, which it contacts by reaching across the Fab domain, completely encircling the antibody.

In addition to these structural differences, MIB exhibits multiple specific characteristics that clearly set it apart from protein M. First, it has the ability to bind to free antibodies as well as to antibodies bound to their antigens. As a result, MIB can be active both pre- and postestablishment of the immune complexes, contrary to protein M, which only acts preemptively. This difference in functionality appears to be linked to the C-terminal domain of protein M, which has to cap the paratope in order for the rest of the protein to properly interact with the antibody light chain. This cap is missing from MIB, thus allowing the protein to bind the antibody even in the presence of the antigen. Second, MIB also has the unique property of being able to dissociate the antibody-antigen complex by inducing large conformational changes of the V_H_ and V_L_ domains, leading to a complete disruption of the antigen-binding site (76). This conformation shift also appears to be important for the recruitment of MIP, as a natively placed V_H_ domain would sterically hinder the protease from binding to the antibody.

From an evolutionary standpoint, MIB appears to be a distant ancestor of protein M. Protein M and MIB are mutually exclusive, and MIB is found in the majority of animal-pathogenic *Mollicutes*, while protein M is restricted to a small number of species, all belonging to the *Pneumoniae* group (Fig. 3). In this group, it is intriguing to note that the closely related species M. genitalium and *Ureaplasma* spp., which share the same host (human) and tissue tropism (urogenital tract) have each selected a different IBP system. It is also noteworthy that the transition from MIB to protein M has been accompanied by the loss of MIP. It is also of interest to note that the vast majority of *Mollicutes* species have an IBP system, with a few exceptions such as Mycoplasma mobile (fish pathogen), Mycoplasma fermentans (human pathogen), and Mycoplasma columbinasale (pigeon pathogen). Currently, the driving forces behind the selection of a given IBP system, or no IBP system, are unknown, and they appear to be uncorrelated with the host species.

## PUTATIVE FUNCTIONS OF MYCOPLASMA IMMUNOGLOBULIN-BLOCKING PROTEINS

The sophisticated strategies deployed by mycoplasmas to directly target antibodies appear to be key components of their interactions with the host. Both protein M and MIB offer mycoplasmas an option to prevent antigen binding, a critical process of the immune system. At the time of writing, the impact of these strategies during infection has not yet been measured in animal models, and their exact roles are not known. However, given their function, they might be involved in multiple phases of infection.

First, these effectors could be key for initial colonization. Indeed, the first layer of host defenses in the mucosa is immune exclusion, a process based on a diverse pool of polyreactive secretory immunoglobulin A (sIgA) natural antibodies (77–80). These low- to medium-affinity immunoglobulins are found in most vertebrates and are continuously produced even in the absence of immunization. They can bind to a multitude of foreign antigens and trap them in the regularly cleared mucus layer. This mechanism has to be bypassed by mycoplasmas in order to reach, colonize, and persist at the surface of the epithelial cells. In addition, sIgA appears to be involved in blocking foreign organisms from binding to mucosal surfaces by neutralizing their adhesins (81). Therefore, mycoplasma IBPs could act as “adhesins protectors” by preventing antibody binding or by clearing bound antibodies.

Second, proteolytic degradation of immunoglobulins by MIB-MIP could be highly relevant to the question of mycoplasmas pathogenesis. Indeed, the hydrolyzed antibodies resulting from the processing by MIB-MIP are likely to be detected by the innate immunity activator leukocyte immunoglobulin-like receptor 2 (LILRA2) (82, 83). This protein is part of the LILR superfamily of paired receptors, expressed at the surface of human myeloid cells and regulating the immune system (84). LILRA2 is an orphan activating receptor and has the ability to recognize cryptic epitopes on the antibody light chain that are normally covered by the V_H_ domain. Upon binding to its ligand, LILRA2 acts as an activator of the nuclear factor of activated T cells (NFAT) pathway, leading to production of the proinflammatory mediator interleukin-8 (IL-8). Through its action as a signaling molecule, IL-8 fosters the recruitment of neutrophils to the lung epithelium. These findings are convergent with the well-established neutrophil accumulation in lungs during infections by respiratory mycoplasmas (85–89). Neutrophils counteract the infection through phagocytosis and/or the release of neutrophil extracellular traps (NETs) (90). Interestingly, mycoplasma lipoproteins have been shown to be major stimulators of NET secretions (86, 91), and some *Mycoplasma* species appear to have dedicated effectors to evade the NETs (91, 92). Overstimulation of neutrophils has been shown to result in further release of proinflammatory molecules and protease, causing damage of lung tissue (93). Furthermore, it was also demonstrated that human neutrophils expressing LILRA2 produce a large oxidative burst in the presence of antibodies lacking their V_H_ domain. The LILRA2/MIB-MIP interplay could provide an explanation for the inflammatory nature of most mycoplasma disease (8), with antibody degradation being one of the root causes of the hyperstimulation of the innate immune response. In addition to human, families of genes encoding LILR receptors have been identified in murine, caprine, bovine, swine, and avian species (94–98). Characterization of these receptors is ongoing, and the human LILRA2 function has not yet been demonstrated in other species, although it is highly probable.

Third, direct targeting of antibodies is probably an important factor in mycoplasma persistence. The humoral immune response to mycoplasma infections appears to be typical, first with the production of high titers of high-affinity IgM followed by class switching to IgG and IgA (15). Our findings have confirmed that MIB-MIP can indeed process and disable high-affinity antibodies targeting surface antigens and is involved in preventing agglutination by IgM (76). It is, however, unclear whether this system is potent enough to counter the large amounts of specific antibodies generated during the immune response or if it is eventually saturated and overwhelmed. The localization of mycoplasmas at the surface of the epithelial layer might mitigate this factor, as antibody concentrations are typically lower in this tissue than in serum (99). Furthermore, the specific antibody titers tend to progressively decrease after the postinfection peak. Following this decay, the antibody concentration could drop below a threshold at which it no longer saturates the IBP systems, allowing the pathogen to reemerge (either from an internal population of evaders hidden in cells, in specific tissues, or from an external reservoir).

Finally, antibody degradation could be an important factor in establishing the multipathogenic complex diseases that are frequently associated with mycoplasmas. It is currently unknown whether mycoplasma infections are subsequent to other pathogens’ colonization, if they precede it, or if they are simultaneous. Interestingly, two studies serendipitously evidenced the generalized degradation of immunoglobulins by mycoplasmas in contaminated cell cultures. In 1980, Dickson et al. reported that Mycoplasma orale, Mycoplasma arginini, and Mycoplasma hyorhinis were able to abrogate the neutralization of vesicular stomatitis virus particles by sheep antiserum (100). The origin of this activity was not identified at the time, but the authors proposed that “the mycoplasma could excrete or have on its surface proteases […] which are capable of destroying the antigen-antibody complexes.” In 2016, Hirayasu et al. reported that M. hyorhinis was able to cleave off the V_H_ domain of nonspecific IgM, IgA, and IgG, probably through MIB-MIP, with up to 50% of the antibodies present in the culture media being degraded (82). Based on these data, we could hypothesize that mycoplasma colonization effectively depletes the pool of antibodies in the mucosa, creating a *de facto* local immune deficiency, which facilitates the infection by other pathogens.

## TAKING MYCOPLASMAS’ ANTI-IMMUNOGLOBULIN STRATEGIES INTO ACCOUNT TO IMPROVE CONTROL STRATEGIES

Given the potentially critical role of anti-immunoglobulin strategies in mycoplasma infections, they should be taken into consideration when planning for future studies on pathogenesis and vaccine development.

First, efforts should be made to better characterize the antibody response to mycoplasmas. While most studies of natural or experimental mycoplasma infection report the titers of pathogen-specific antibodies in the serum, this metric might not be representative of the immune pressure on the pathogens at the infection site. Indeed, both titers and composition of the antibody pool have been shown to vary drastically between the systemic and mucosal compartments, in particular, in the lungs. While it is understood that sampling serum is guided by ease of sample collection, lavages of infected tissues should also be performed and their antibody content analyzed. Furthermore, the classes (especially IgG, IgM, and sIgA) and subclasses of pathogen-specific immunoglobulins should be systematically titrated, as they can be targeted in a differential manner by bacterial effectors and their involvement in the immune response has been shown to vary over the course of long infections. In addition to titration, we should also strive to identify which bacterial antigens are targeted in order to evaluate the richness and diversity of the humoral response. Recently developed high-throughput epitope-mapping strategies (101) such as phage immunoprecipitation sequencing (PhIp-Seq) allow efficient mapping of the antibody repertoire and should be considered and adapted to relevant mycoplasma species and broadly adopted in infection studies. Such tools could also be invaluable to study the hypothesized impact of mycoplasmas on coinfection emergence by depletion of the native antibody repertoire.

Second, the time frame in which mycoplasma infections are studied should be expanded significantly, potentially by 1 order of magnitude. Indeed, due to the cost of animal husbandry and ethics requirements regarding animal suffering and euthanasia, most studies duration range from a few days to a few months depending on the *Mycoplasma* species and the animal model. Increasing the duration to a year or more, in particular, in larger animals, will be necessary to better understand the interplay between chronic episodes during mycoplasma infections and the fluctuation of specific antibody titers. This remark can also be applied in the frame of vaccine development, as we might need to reevaluate the time point at which vaccinated animals are challenged. In most cases, challenges occur 30 to 60 days after immunization, when antibody titers are still high. Although this short duration might be adequate for animals with short lifespans (broiler poultry, finishing pigs), it does not appear to be suitable for animals farmed over longer, often multiyear cycles (beef cattle or dairy cows and goats).

Finally, the discovery of mycoplasma IBPs should lead to a reevaluation of the approaches taken toward vaccine development. For instance, vaccines that are based on recombinant proteins have yet to be successful against mycoplasmas. We would argue that this is unsurprising given the shield that is provided by the MIB-MIP system. Indeed, the saturation of this defense system is probably more difficult to obtain with a vaccine that only contains one or a few protein antigens. It can be argued that a recombinant vaccine targeting directly MIB and/or MIP could potentially be developed to solve this issue. However, given the high sequence variability of these proteins and the frequent co-occurrence of multiple paralogs, this prospect seems arduous. Conversely, inactivated or attenuated vaccines have the advantage of presenting a wide array of antigens, including MIB-MIP. Some attenuated vaccine strains of mycoplasmas are used in ruminants and poultry, although the protection that is provided is usually short-lived. It is unclear whether the MIB-MIP system is still functional in these strains and if its presence has an impact on vaccine efficacy.

## CONCLUDING REMARKS

Mycoplasmas are characterized by small genomes and are therefore often viewed as simple organisms. Interestingly, it seems that they have evolved elaborate and independent strategies to cope with the selective pressure applied by their hosts’ immune systems, making the most of their quasiminimal genetic information. Since the 1980s, mycoplasmologists have documented the mechanisms leading to an ever-changing surface antigenic structure of mycoplasma cells. These studies have shaped the view that stochastic events provide these organisms with the means to escape immune surveillance and to persist either as commensals or as chronic infectious agents. More recently, the discoveries of the new mycoplasma-specific IBPs protein M and “MIB” have completely renewed our view of mycoplasma immune evasion. Indeed, they correspond to active mechanisms that directly target the host antibodies by preventing their interactions with the antigens, thus negating a critical component of the immune response. The fact that either protein M or the MIB-MIP system can be found in the vast majority of animal-infecting mycoplasmas suggests a key role of these effectors in dealing with the host’s antibodies. At the moment, we still lack information about the role of these IBPs *in vivo*. Their characterization does, however, have a number of practical implications, in particular, regarding vaccine developments. It appears essential to understand how the production of specific antibodies can overcome the protection provided by the MIB-MIP system and the kinetics of this interaction. There is also a renewed interest in understanding the potentially immunosuppressive role that the MIB-MIP system could play in setting the ground for other concomitant infections.
